# 146例先天性纤维蛋白原病的基因突变谱及纤维蛋白原输注药代动力学分析

**DOI:** 10.3760/cma.j.issn.0253-2727.2021.07.005

**Published:** 2021-07

**Authors:** 丽颖 黄, 冬雷 张, 荣凤 付, 葳 刘, 云飞 陈, 峰 薛, 晓帆 刘, 婷婷 毕, 仁池 杨, 磊 张

**Affiliations:** 中国医学科学院、北京协和医学院血液病医院（中国医学科学院血液学研究所），实验血液学国家重点实验室，国家血液系统疾病临床医学研究中心，天津市血液病基因治疗研究重点实验室，中国医学科学院血液病基因治疗重点实验室，天津 300020 State Key Laboratory of Experimental Hematology, National Clinical Research Center for Blood Diseases, Tianjin Laboratory of Blood Disease Gene Therapy, CAMS Key Laboratory of Gene Therapy for Blood Diseases, Institute of Hematology & Blood Diseases Hospital, Chinese Academy of Medical Sciences & Peking Union Medical College, Tianjin 300020, China

**Keywords:** 先天性纤维蛋白原缺陷症, 基因突变, 热点突变, 临床表型, Congenital fibrinogen disorders, Gene mutation, Hot spot mutation, Clinical manifestation

## Abstract

**目的:**

探讨先天性纤维蛋白原病的基因突变类型与分型、临床表现、实验室检查、诊断及纤维蛋白原替代治疗情况。

**方法:**

对2003年4月至2020年11月就诊于中国医学科学院血液病医院的146例先天性纤维蛋白原病患者进行回顾性分析。

**结果:**

146例患者中，男61例（41.8％），女85例（58.2％），就诊时中位年龄为33.5岁；共采集到98例患者的临床症状学信息，34例（34.7％）因出血症状而就诊，33例（33.7％）因手术前检查而确诊，55例（56.1％）患者至少有1次出血，42例（42.9％）无出血表现。纤维蛋白原活性（FIB∶C）与ISTH-BAT出血评分之间呈负相关（*rs*＝−0.412，*P*<0.001）。在56例患者中检出34种基因突变（包括新突变16种），其中84.1％为错义突变，FGA外显子2和FGG外显子8突变占全部突变位点的71.4％。无纤维蛋白原血症患者（7例）中位年龄为2（1～12）岁，ISTH-BAT评分为4分；异常纤维蛋白原血症患者（50例）中位凝血酶时间为28.5（19.2～36.6）s。输注纤维蛋白原后1 h、24 h的活性回收率（IVR）分别为（127.19±44.03）％、（101.78±43.98）％。输注纤维蛋白原后，FIB∶C明显提升，凝血酶时间及凝血酶原时间明显下降，用药24 h后凝血酶时间较基线平均下降（15.2±12.1）％。

**结论:**

多数先天性纤维蛋白原缺乏症患者症状轻微或无症状；无纤维蛋白原血症患者出血症状较为严重，FIB∶C与出血程度之间存在负相关；基因检测有助于疾病分型诊断；FIB∶C/纤维蛋白原抗原（FIB∶Ag）比值<0.7可作为临床分型依据。凝血酶时间可作为异常纤维蛋白原血症诊断及纤维蛋白原替代治疗的疗效判定依据。

## Abstract (en)

**Objective:**

To investigate the clinical type and gene mutations, clinical manifestations, laboratory tests, diagnosis, and fibrinogen replacement therapy of congenital fibrinogen disorders.

**Methods:**

Clinical data of 146 patients with congenital fibrinogen disorders diagnosed from April 2000 to November 2020 were retrospectively analyzed.

**Results:**

Among the 146 patients, 61（41.8％）men and 85（58.2％）women had a median age of 33.5 years at the time of consultation. 34 patients（34.7％）were found to suffer from the disease due to bleeding symptoms, 33 patients（33.7％）due to preoperative examination. 55 patients（56.1％）had at least one bleeding symptom, and 42 patients（42.9％）had no bleeding symptoms. There is a negative correlation between fibrinogen activity concentration and bleeding ISTH-BAT score（rs＝−0.412, *P*＝0.001）. A total of 34 gene mutations were detected in 56 patients, of which 84.1％ were missense mutations, and 16 new mutations were found. FGA Exon2 and FGG Exon8 mutations accounted for 71.4％ of all mutation sites. Patients with afibrinogenemia were younger, with a median age of 2（1–12）years, an ISTH-BAT score of 4, and patients with dysfibrinogenemia had significantly longer thrombin time（TT）, with a median of 28.5（19.2–36.6）s. The 1 hour in vivo recovery（IVR）after fibrinogen infusion was（127.19±44.03）％, and the 24 hour IVR was（101.78±43.98）％. In addition to the obvious increase in the concentration of fibrinogen activity, the TT and the prothrombin time（PT）both decreased significantly, and the TT decreased more significantly, with an average decrease of 15.2％ compared to the baseline after 24 hours of infusion.

**Conclusion:**

Most patients with congenital fibrinogen disorders have mild or no bleeding symptoms. Patients with afibrinogenemia have more severe symptoms. There is a negative correlation between the fibrinogen and the degree of bleeding. Genetic testing is helpful for the diagnosis of disease classification. FIB∶C/FIB∶Ag<0.7 can be used as a basis for clinical diagnosis. The TT can be used as the basis for the diagnosis of dysfibrinogenemia and the effectiveness of fibrinogen infusion.

纤维蛋白原是由二硫键将两个相同的异质三联体连接而成的六聚体，每个异质三联体包含1条Aα链、1条Bβ链和1条γ链，编码基因分别为FGA、FGB、FGG[1]。先天性纤维蛋白原缺乏症是一种由基因缺陷而导致纤维蛋白原含量或（和）功能异常的疾病，其中Ⅰ型缺陷表现为纤维蛋白原量的缺乏（根据纤维蛋白原含量降低的程度又分为无纤维蛋白原血症和低纤维蛋白原血症），Ⅱ型缺陷为纤维蛋白原质的异常（包括异常纤维蛋白原血症和低异常纤维蛋白原血症）[2]。先天性纤维蛋白原病是一种罕见的出血性疾病，无纤维蛋白原血症的年发病率约为1/10万[3]。世界血友病联盟调查发现纤维蛋白原缺乏症占所有罕见出血性疾病的8％，相对于其他常染色体遗传性疾病，其发病率呈上升趋势[4]。本研究对就诊于中国医学科学院血液病医院的146例先天性纤维蛋白原病患者进行回顾性分析，以探讨先天性纤维蛋白原病的临床表现、实验室检查、诊断、基因突变及纤维蛋白原替代治疗情。

## 病例与方法

1. 病例：本研究纳入2003年4月至2020年11月于中国医学科学院血液病医院就诊的146例先天性纤维蛋白原病患者，共采集到98例患者的临床症状学信息。采用ISTH-BAT标准[5]进行出血评分。

2. 方法：所有患者均进行凝血功能检测，包括活化部分凝血活酶时间（APTT）、凝血酶原时间（PT）、凝血酶时间（TT）、纤维蛋白原活性（FIB∶C）和纤维蛋白原抗原（FIB∶Ag）。FIB∶C检测采用Clauss法，FIB∶Ag检测采用免疫比浊法。根据同批次标本测得的FIB∶C/FIB∶Ag比值对患者进行诊断分型：FIB∶C/FIB∶Ag比值<0.7且FIB∶Ag正常，诊断为异常纤维蛋白原血症；FIB∶C/FIB∶Ag比值<0.7但FIB∶Ag低于正常，诊断为低异常纤维蛋白原血症；FIB∶C/FIB∶Ag比值>0.7，诊断为低/无纤维蛋白原血症[2],[6]–[7]；APTT、PT、TT明显延长或标本不凝无法测得结果，无法检测或者仅检测到水平极低的纤维蛋白原（<0.1 g/L），诊断为无纤维蛋白原血症[1]。

对进行纤维蛋白原输注的患者进行活性回收率（IVR）计算，具体公式如下[8]：

$$\text{活性回收率（％）}=\frac{\text{治疗后纤维蛋白原活性}-\text{治疗前纤维蛋白原活性}}{\text{预计提升的纤维蛋白原活性}}\times 100\%$$

$$\text{预计提升的纤维蛋白原活性（g/L）}=\frac{\text{输注剂量（g）}}{0.07}\times \text{体重（kg）}$$

3. 靶向测序检测基因突变：采取样本细胞基因组DNA，分析基因蛋白质编码区域及外显旁侧内含子区的点突变和短片段插入/缺失突变。对目的基因序列采用脱氧核糖核酸测序检测突变位点。测序后原始数据利用1000genomes、SIFT、PolyPhen-2、Mutationtaster等数据库进行生物信息学分析。

4. 统计学处理：定量资料数据以“中位数（范围）”或“均数±标准差”表示，定性资料数据以数量、百分比表示。用shapiro-wilk检验数据正态分布，对于不符合正态分布的数据，采用spearman相关分析两个连续性变量之间的关系，采用Kruskal-Wallis *H*检验来分析不同组间的数据差异。分类变量资料组间的比较采用Fisher精确概率法。采用配对*t*检验来比较治疗前后的凝血功能变化。采用SPSS 21.0软件对数据进行统计学处理，以*P*<0.05为差异有统计学意义。使用Origin软件作图。

## 结果

1. 临床特征：本研究纳入2003年4月至2020年11月于我院就诊的146例先天性纤维蛋白原病患者。男61例（41.8％），女85例（58.2％），中位就诊年龄为33.5（1～76）岁。98例患者具有临床症状学信息，其中19例（19.4％）有家族史，34例（34.7％）因出血症状而就诊，33例（33.7％）因手术前检查发现，9例（9.2％）因家族成员确诊而发现，9例（9.2％）为健康查体确诊，6例（6.1％）为外伤后发现，5例（5.1％）患者因其他疾病就医而发现，2例（2.0％）因血栓症状确诊。在有症状信息的98例患者中，55例（56.1％）至少有1次出血症状，42例（42.9％）无任何出血症状，1例（1.0％）表现为下肢静脉血栓。在55例有出血症状的患者中，23例（41.8％）存在皮肤瘀点，15例（27.3％）有牙龈出血，8例（14.6％）有鼻出血，6例（10.9％）有消化道出血（包括2例痔疮出血），5例（9.1％）有月经延长，3例（5.5％）发生颅内出血，2例（3.6％）出生时有脐带残端出血，2例（3.6％）有关节肌肉出血，2例（3.6％）有阴道出血，2例（3.6％）分娩时出血增多。

2. 实验室检查：主要表现为FIB∶C降低和TT延长。146例患者的初诊凝血功能检测结果（中位数）：FIB∶C 0.66（0～1.86）g/L，APTT 28.2（18.1～47.5）s，TT 25.7（16.3～53.1）s，PT 12.9（9.2～22.6）s，无纤维蛋白原血症患者的凝血功能显示不凝，未计入在内。在有完整临床病历记录的98例患者中，平均FIB∶C为（0.68±0.47）g/L，平均ISTH-BAT出血评分为（1.40±1.52）分，由于shapiro-wilk检验显示ISTH-BAT评分不符合正态分布，故采用spearman相关分析评价FIB∶C与ISTH-BAT出血评分之间的关系。结果显示，FIB∶C与ISTH-BAT评分呈负相关（*rs*＝−0.412，*P*<0.001），FIB∶C低于0.5 g/L时，患者的出血症状较为明显，FIB∶C>1 g/L时出血较轻（图1）。

**图1:**
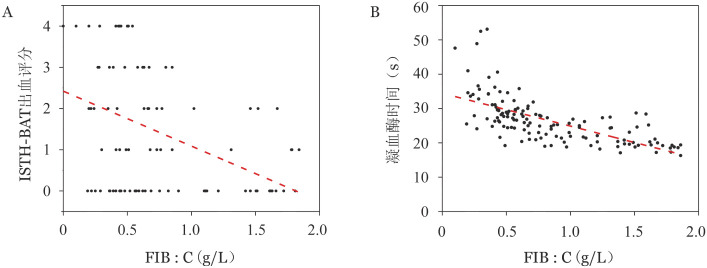
纤维蛋白原活性（FIB∶C）、ISTH-BAT出血评分（A）与凝血酶时间（B）的相关性分析

3. 疾病分型：我们根据患者FIB∶C、FIB∶Ag以及基因突变表型，共确诊7例患者为无纤维蛋白原血症、29例患者为低纤维蛋白原血症、50例患者为异常纤维蛋白原血症、11例患者为低异常纤维蛋白原血症。无纤维蛋白原血症组的中位年龄为2（1～12）岁，低纤维蛋白原血症组的中位年龄为24（1～76）岁，异常纤维蛋白原血症组的中位年龄为34（5～68）岁，低异常纤维蛋白原血症患者的中位年龄为38（31～76）岁。采用Kruskal-Wallis *H*分析，无纤维蛋白原血症患者与其他三组患者相比年龄较小（*H*＝25.714，*P*<0.001），低纤维蛋白原血症患者较低异常纤维蛋白原血症患者年龄小（*P*＝0.017），其他组间差异无统计学意义。

低纤维蛋白原血症的中位TT为21.1（16.3～48.9）s，异常纤维蛋白原血症的中位TT为28.5（19.2～36.6）s，低异常纤维蛋白原血症患者的中位TT为24.8（20.4～34.6）s（因无纤维蛋白血症患者的凝血功能显示不凝，故未纳入比较）。异常纤维蛋白原血症患者的TT较低纤维蛋白原血症患者明显延长（*H*＝34.299，*P*<0.001），其他组间比较差异无统计学意义。低纤维蛋白原血症、异常纤维蛋白原血症、低异常纤维蛋白原血症患者的中位FIB∶C分别为1.00（0.27～1.86）g/L、0.54（0.19～1.61）g/L、0.51（0.20～1.07）g/L，异常纤维蛋白原血症、低异常纤维蛋白原血症患者的FIB∶C明显低于低纤维蛋白原患者（*H*＝27.924，*P*<0.001；*H*＝29.882，*P*＝0.026），而异常与低异常纤维蛋白原血症之间无显著性差异（*P*＝1.000）。此外异常纤维蛋白原血症患者的FIB∶Ag高于另外两组患者（详见表1）。

**表1:** 97例先天性纤维蛋白原病患者的疾病表型

项目	无纤维蛋白原血症（7例）	低纤维蛋白原血症（29例）	异常纤维蛋白原血症（50例）	低异常纤维蛋白原血症（11例）
性别（例，男/女）	3/4	15/14	16/34	2/9
年龄［岁，*M*（范围）］	2（1～12）	24（1～76）	34（5～68）	38（31～76）
基因突变［例（％）］				
FGA	5（71.4）	1（14.3）	22（57.9）	2（33.3）
FGB	2（28.6）	4（57.1）	2（5.3）	0（0）
FGG	0（0）	2（28.6）	14（36.8）	4（66.7）
ISTH-BAT评分［例（％）］				
0	0（0）	6（50.0）	23（56.1）	3（50.0）
1	0（0）	2（16.7）	6（14.6）	1（16.7）
2	0（0）	0（0）	4（9.8）	1（16.7）
3	0（0）	2（16.7）	4（9.8）	1（16.7）
4	7（100.0）	2（16.7）	4（9.8）	0（0）
初诊APTT［s，*M*（范围）］	–	28.9（23.1～38.3）	28.0（21.9～47.5）	29.1（24.6～36.7）
初诊TT［s，*M*（范围）］	–	21.1（16.3～48.9）	28.5（19.2～36.6）	24.8（20.4～34.6）
初诊PT［s，*M*（范围）］	–	12.6（10.3～19.3）	13.2（11.0～16.8）	12.9（11.1～17.8）
初诊FIB∶C［g/L，*M*（范围）］	–	1.00（0.27～1.86）	0.54（0.19～1.61）	0.51（0.20～1.07）
初诊FIB∶Ag［g/L，*M*（范围）］	–	1.31（0.10～2.56）	2.53（1.90～8.98）	1.17（0.79～1.79）

注：APTT：活化部分凝血活酶时间；PT：凝血酶原时间；TT：凝血酶时间；FIB∶C：纤维蛋白活性；FIB∶Ag：纤维蛋白原抗原；–：不凝

无纤维蛋白原血症组的ISTH-BAT评分均为4分，其中有4例患者出生时发生脐带残端出血，4例患者发生鼻出血且不易止血，2例患者发生颅内出血，2例患者存在关节肌肉内血肿，1例患者出现广泛帽腱膜下出血。Fisher精确检验显示，无纤维蛋白原血症患者的BAT评分明显高于其他三组患者（*P*＝0.003）。低纤维蛋白原血症、异常纤维蛋白原血症、低异常纤维蛋白原血症的BAT评分均值分别为1.33±1.67、1.10±1.41、1.00±1.26，三组之间差异无统计学意义（*P*＝1.000），且超60％的患者的BAT评分为0～1分。此外，异常及低异常纤维蛋白原血症患者常见FGA和FGG突变，而FGB基因突变更常见于无或者低纤维蛋白原血症患者中（表1）。

4. 基因突变：有56例患者进行了基因检测，其中7例检出多个位点突变，共检测到63例突变，不同类型的基因突变有34种（FGA基因突变17种，FGB基因突变4种，FGG基因突变13种，详见表2）。查阅相关文献及HGMD、Clinvar数据库以及https://site.geht.org/base-de-donnees-fibrinogene/网站，共确认其中16种为新发突变。有23例（41.07％）基因突变位点位于FGA基因的第2外显子（Exon2），有17例（30.35％）基因突变位点位于FGG基因第8外显子，即FGA外显子2和FGG外显子8突变占全部突变位点的71.4％。在这些突变中，错义突变53例（84.1％），移码突变5例（7.9％），无义突变4例（6.4％），剪接位点突变1例（1.6％）。其中频繁出现的热点突变为FGGArg301His（c.902G>A）、FGG Arg301Cys（c.901C>T）、FGA Arg35His（c.104G>A）和FGA Arg35Cys（c.103C>T）。

**表2:** 纤维蛋白原缺乏症患者的基因突变表型

突变基因	染色体位置	转录本ID	突变位置	核苷酸改变	氨基酸改变
FGA	chr4:155510663	NM_000508	Exon2	c.G106A	p.G36S
FGA	chr4:155510657	NM_000508	Exon2	c.112A>G	p.R38G
FGA	chr4:155510655	NM_000508	Exon2	c.114G>C	p.R38S
FGA	chr4:155510686	NM_000508	Exon2	c.83T>C	p.L28P
FGA	chr4:155510666	NM_000508	Exon2	c.103C>T	p.R35C
FGA	chr4:155510665	NM_000508	Exon2	c.104G>A	p.R35H
FGA	chr4:155510606	NM_000508	Exon2	c.163T>C	p.C55R
FGA	chr4:155510689	NM_000508	Exon2	c.80T>C	p.F27S
FGA	chr4:155510675	NM_000508	Exon2	c.94G>A	p.G32R
FGA	chr4:155510117	NM_000508	Exon3	c.192C>A	p.C64X
FGA^a^	chr4:155507063	NM_000508	Exon5	c.1517delT	p.L506fs
FGA	chr4:155509980	NM_000508	Exon3	c.T329C	p.M11T
FGA^a^	chr4:155505649	NM_000508	Exon6	c.G2228A	p.R743Q
FGA	chr4:155508672	NM_000508	Exon4	c.502C>T	p.R168X
FGA^a^	chr4:155507913	NM_000508	Exon5	c.668delT	p.L223Rfs*2
FGA	chr4:155508726	NM_000508	Exon4	c.448C>T	p.Q150X
FGA^a^	chr4:155507843	NM_000508	Exon5	c.737dupC	p.E247Rfs*22
FGB^a^	chr4:155490951	NM_005141	Exon7	c.1244G>T	p.W415L
FGB^a^	chr4:155488786	NM_005141	Exon4	c.537_568dup	p.N190Tfs*5
FGB	chr4:155490354	NM_005141	Exon6	c.853C>T	p.R285C
FGB^a^	chr4:155488764	NM_005141	Exon4	c.510T>A	p.N170K
FGG^a^	chr4:155531251	NM_000509	Exon5	c.A500G	p.D167G
FGG	chr4:155528084	NM_000509	Exon8	c.902G>A	p.R301H
FGG^a^	chr4:155527935	NM_000509	Exon8	c.1051A>G	p.N351D
FGG^a^	chr4:155527893.0	NM_000509	Exon8	c.1093T>G	p.C365G
FGG	chr4:155528085	NM_021870	Exon8	c.901C>T	p.R301C
FGG	chr4:155527994	NM_021870	Exon8	c.992C>A	p.T331K
FGG^a^	chr4:155528109	NM_021870	Exon8	c.877G>A	p.V293M
FGG	chr4:155527886	NM_021870	Exon8	c.1100C>T	p.A367V
FGG	–	–	Exon8	c.1064A>G	p.Q355R
FGG^a^	chr4:155526137	NM_000509	Exon9	c.1211C>T	p.S404F
FGG^a^	chr4:155526134	NM_021870	Exon9	c.1214T>C	p.M405T
FGG	–	NM_000509	Exon9	c.1185G>A	p.W395*
FGG^a^	chr4:155526219	NM_021870	Intron8	c.1130-1G>T	–

注：–：未获取相关数据；^a^新突变；Exon：外显子

34例患者同时进行基因检测和抗原检测，其中32例（94.12％）符合FIB∶C/FIB∶Ag比值<0.7的标准，有2例（5.88％）患者不符合。

我们共确诊了7例无纤维蛋白原血症患者，其中4例进行了基因检测，基因突变分别为FGB c.537_568dup p.N190Tfs*5、c.853C>T p.R285C；FGA c.502C>T p.R168X、c.668delT p.L223Rfs*2；FGA c.448C>T p.Q150X、c.737dupC p.E247Rfs*22；FGA c.192C>A p.C64X。

5. 家系分析：本研究中有19例患者存在阳性家族史，其中一个遗传性异常纤维蛋白原血症家族7名成员基因检测结果见图2。先证者（Ⅱ-7），女，46岁，9年前因乳腺癌手术前检查发现纤维蛋白原（0.4～0.5）g/L，予新辅助化疗4次后行乳腺癌根治术，术中、术后出血情况正常。凝血功能：APTT 21.9 s、PT 25.3 s、TT 11 s、FIB∶C 0.899 g/L、FDP<2 mg/L、D二聚体<0.19 mg/L，基因检测发现存在FGA外显子2 c.112A>G p.R38G和FGB外显子7 c.1244G>T p.W415L杂合突变。其家族中，Ⅲ-1平素偶有齿龈出血、Ⅲ-2外伤后伤口愈合慢，其他成员无出血表现；11名家族成员于我院行凝血功能检测，Ⅱ-7、Ⅱ-9、Ⅲ-1、Ⅲ-2、Ⅲ-5、Ⅲ-6检出HIB∶Ag下降，Ⅱ-3、Ⅱ-5、Ⅲ-3、Ⅲ-4凝血功能正常。基因检测发现，Ⅱ-9基因检测结果与其一致，均存在上述两个位点突变，其姐（Ⅱ-1）姐姐的两个女儿（Ⅲ-1、Ⅲ-2）以及患者的子女（Ⅲ-5、Ⅲ-6）均只存在FGA外显子2 c.112A>G p.R38G一个杂合突变位点。其另外两个姐姐（Ⅱ-3、Ⅱ-5）以及姐姐的儿子（Ⅲ-3、Ⅲ-4）纤维蛋白原检测正常，未行基因突变检测，Ⅲ-7、Ⅰ-1和Ⅰ-2未行检测，具体情况未知。

**图2:**
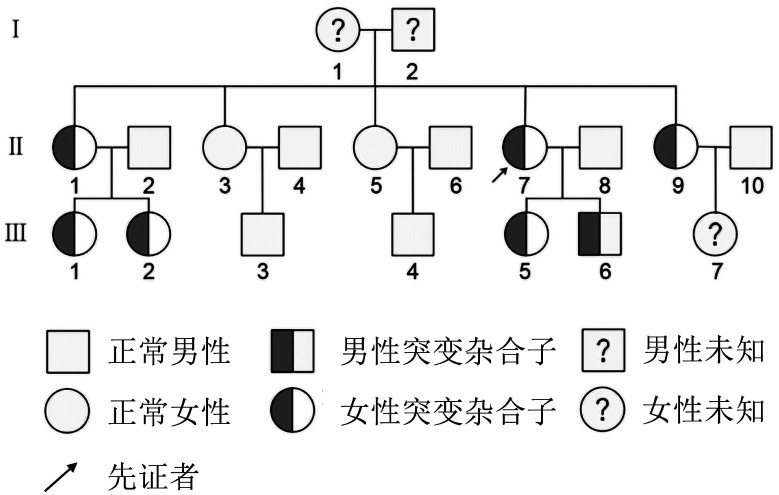
遗传性异常纤维蛋白原血症家系图

6. 纤维蛋白原替代治疗：根据输注纤维蛋白原前、输注后1 h及输注后24 h后均有凝血功能记录的标准，筛选出28例患者共39次纤维蛋白原输注记录。在输注纤维蛋白原前的FIB、APTT、TT、PT检测结果见表3。采用配对*t*检验来比较输注前后患者的凝血功能变化。输注后1 h纤维蛋白原平均升幅为（0.47±0.27）g/L（*t*＝9.873，*P*<0.001），输注24 h后平均升幅为（0.41±0.26）g/L（*t*＝9.732，*P*<0.001），输注后24 h较输注后1 h，患者体内纤维蛋白原含量平均降幅为（0.12±0.14）g/L。输注后1 h的IVR为（127.19±44.03）％（95％ *CI* 111.58％～142.80％），输注后24 h的IVR为（101.78±43.98）％（95％*CI* 87.33％～116.24％）。另外，我们比较了输注1 h以及输注24 h的APTT、TT以及TT较基线的变化，PT及TT值均较基线值明显缩短，TT在用药24 h后较基线下降（15.2±12.1）％（*t*＝5.254，*P*<0.001），而PT在用药24 h后较基线下降（6.27±8.24）％（*t*＝3.675，*P*＝0.001）。

**表3:** 纤维蛋白原替代治疗前后凝血指标变化

项目	输注前	输注后1h	输注后24h	输注后1h与输注前比较		输注后24h与输注前比较	
				*t*值	*P*值	*t*值	*P*值
FIB∶C（g/L）	0.60±0.28	1.08±0.34	1.00±0.29	9.873	<0.001	9.732	<0.001
APTT（s）	27.82±3.50	27.78±2.97	27.44±2.83	−1.834	0.077	1.130	0.266
TT（s）	27.44±5.31	23.82±2.96	23.38±3.76	8.844	<0.001	5.254	<0.001
PT（s）	13.65±2.32	12.88±0.95	12.95±2.09	4.731	<0.001	3.675	0.001

注：FIB∶C：纤维蛋白原活性；APTT：活化部分凝血活酶时间；TT：凝血酶时间；PT：凝血酶原时间

## 讨论

在本研究中，我们共采集到98例患者的临床信息，有55例（56.1％）患者至少有1次出血，这与Anetta等[9]报告的一项为期60个月的随访出血发生率（56％）相当，高于Casini等[10]报告的出血发生率（47.5％）。最常见的出血症状为皮肤瘀点、齿龈出血和鼻出血，但西方国家中还常见女性月经增多[10]。我们研究发现很少有血栓症状，这与以前的国内报道[11]一致。同时，我们发现FIB∶Ag与BAT评分之间存在负相关关系，FIB∶Ag水平越低出血风险越高（出血症状在FIB∶Ag低于0.5 g/L时较为明显）。Peyvandi等[12]也证实了纤维蛋白原水平与临床严重程度之间存在良好的相关性。以往研究结果显示，小手术前FIB∶Ag维持1 g/L，大手术前维持1.5 g/L就足以维持止血[13]，本研究结果显示，FIB∶Ag达到1 g/L即足以预防出血。

本研究中，纤维蛋白原替代治疗后1、24 h的IVR分别为（127.19±44.03）％、（101.78±43.98）％。Kreuz等[8]报告的小队列研究IVR为44.3％～63.8％，后来他们又报道8例接受纤维蛋白原替代治疗患者的IVR为59.8％（32.5％～93.9％）[14]。Négrier等[15]报告的中位IVR为94％（79％～110％）。在Peyvandi等[16]进行的多中心前瞻性临床试验中，IVR为61.8％（52.45％～97.43％），t_1/2_为77.1 h。在国际上已公布的五项评估3种纤维蛋白原药代动力学特性的多中心前瞻试验中，半衰期为67.9～82.4 h[13]，临床上可据此推测输注纤维蛋白原后的维持止血的安全时间。此外PT及TT均有下降，且TT反应最为灵敏，24 h后较基线水平下降（15.2±12.1）％，因TT反映凝血共同途径中纤维蛋白原转变为纤维蛋白的过程[17]，纤维蛋白原的补充可直接缩短这一时间，本组病例输注纤维蛋白原后TT缩短5 s，提示TT可作为判断纤维蛋白原输注疗效的指标。

我们根据FIB∶C/FIB∶Ag<0.7诊断为异常或低异常纤维蛋白原血症，FIB∶C/FIB∶Ag>0.7诊断为低或无纤维蛋白原血症。与基因突变检测结果比较，该值有94.12％的准确率，高于Casini等[18]计算的85％的准确率，进一步验证了FIB∶C/FIB∶Ag比值<0.7作为临床分型诊断依据的可靠性。其他研究表明FIB∶C/FIB∶Ag比值<0.7这一比值几乎可以识别几乎所有的异常纤维蛋白原血症[19]，但该临界值的敏感性和特异性均未得到前瞻性研究的验证。本组病例研究结果提示准确的诊断尚需要基因突变检测，基因检测可将准确率提高近6％。

无纤维蛋白原血症极为罕见，呈染色体不完全隐性遗传。本组病例中无纤维蛋白原血症患者年龄较低且出血症状较为严重，表现为脐带出血、颅内出血、鼻腔出血量大不易止、关节肌肉内血肿和广泛帽腱膜下出血。欧洲流行病学调查发现85％的无纤维蛋白原血症患者存在脐带出血，脑出血是其主要死亡原因[20]。在本研究中，无纤维蛋白原血症患者的基因突变多为无义突变和移码突变，其中4例患者中有3例存在多个位点突变。国外文献中报道无纤维蛋白原血症突变类型以大片段缺失、移码突变、以及无义突变为主[21]，这些无效突变可导致纤维蛋白原合成、组装或分泌不良，从而引起纤维蛋白原的极度缺乏[22]，进而导致无纤维蛋白原血症，并且从我们的研究中可推测突变位点的增多可能导致纤维蛋白原缺乏的程度增加。

本组病例中异常及低异常纤维蛋白原血症患者的FIB∶C显著低于低纤维蛋白原血症。Wan等[23]的发现低异常纤维蛋白原血症患者的FIB∶C最低，异常和低纤维蛋白原血症患者的FIB∶C相近。本组病例异常纤维蛋白原血症患者TT显著延长，Roberts等[24]推荐将TT明显延长作为初始诊断异常纤维蛋白原血症的依据。异常以及低异常纤维蛋白原血症患者基因突变以错义突变为主，且FGA外显子2和FGG外显子8的突变占全部突变位点的71％，欧洲的流行病学调查则显示这两个位点的突变占比83.9％[22]，提示对患者进行基因检测时可优先筛查这两个区域以提高效率。我们发现异常及低异常纤维蛋白原血症患者常见FGA和FGG基因突变，无/低纤维蛋白原血症患者常见FGB基因突变，这与Casini等[22]的研究结果一致，他们发现低纤维蛋白原血症70％的错义突变发生于FGB。目前http://site.geht.org/的在线数据库中记录了250多种致病突变，我们在本组病例中发现了16种新突变，中国人群的热点突变FGG Arg301His（c.902G>A）、FGG Arg301Cys（c.901C>T）、FGA Arg35His（c.104G>A）和FGA Arg35Cys（c.103C>T）与欧美国家一致[22]，欧洲国家常见的FGA IVS 4+1[21]在本组病例中没有检出。

此外，基因突变分析可对有家族史的患者进行提前诊断，在妊娠、手术等情况进行替代治疗以防止发生出血[25]。而且基因突变分析可以鉴别先天性纤维蛋白原缺乏和继发性纤维蛋白原缺乏，从而明确病因，有助于指导长期治疗。基因突变分析还可以明确纤维蛋白原结构或者功能的异常，这对诊断异常纤维蛋白原血症尤为重要。此外，基因突变分析还可预测部分患者的临床表型，例如部分FGG突变与遗传性淀粉样变性相关，部分FGA突变与血栓形成相关[26]。因此，我们建议在条件允许的情况下对纤维蛋白原缺乏患者进行基因突变检测。

综上，本组先天性纤维蛋白原病患者多数症状轻微或者无症状，无纤维蛋白原血症患者症状严重且年龄较小，需引起重视。FIB∶C与出血程度存在负相关关系，FIB∶Ag>1 g/L足以预防出血。基因检测有助于疾病分型诊断，FIB∶C/FIB∶Ag比值<0.7可作为临床分型诊断的依据。TT可作为异常纤维蛋白原血症的诊断依据以及纤维蛋白原替代治疗的疗效判定指标。
